# A case of scalp psoriasis resistant to ixekizumab treated with bimekizumab

**DOI:** 10.1016/j.jdcr.2023.05.043

**Published:** 2023-07-03

**Authors:** Matteo Megna, Vincenzo Picone, Virginia Ventura, Fabrizio Martora, Angelo Ruggiero, Gabriella Fabbrocini, Teresa Battista

**Affiliations:** Section of Dermatology, Department of Clinical Medicine and Surgery, University of Naples Federico II, Napoli, Italy

**Keywords:** bimekizumab, biologics, psoriasis

## Introduction

Psoriasis is a chronic systemic inflammatory skin disease that deeply impacts patients’ lives with a chronic-recurrent course.^1^ Globally, it has a prevalence of 3% in the general population, with possible differences between countries.^2^ Scalp involvement is observed in up to 79% of patients with psoriasis.^2^ Scalp psoriasis is a social impediment in approximately 50% of patients, causing significant psychosocial morbidity.^2^ Scalp falls within difficult-to-treat areas, being hardly suitable for topical agents use as well as often showing resistance to conventional treatments.^2^ Several treatment options are available with variable efficacy, such as topical agents (topical steroids, keratolytics, and vitamin D analogs), systemic conventional agents (methotrexate, cyclosporine, fumarates, and oral retinoids), small molecules (oral phosphodiesterases 4 inhibitors), and biologics. In recent years, after the introduction of biologic drugs, a number of more effective alternatives are available for the treatment of scalp psoriasis; however, there is no universally agreed consensus on first-line treatment, and even the new effective biologics, such as anti-interleukin (IL) 17 and IL-23, may experience primary or secondary failure.^3^ Bimekizumab is a novel humanized monoclonal antibody that inhibits both IL-17A and IL-17F.^4^ These 2 ILs have been shown to be functionally dysregulated in lots of immune-mediated inflammatory diseases, such as psoriasis and psoriatic arthritis (PsA).^4^ Based on these assumptions, bimekizumab was developed to provide a better clinical response by blocking both IL-17A and IL-17F than by inhibiting IL-17A alone with secukinumab or ixekizumab.^4^ It was recently approved for the treatment of moderate-to-severe plaque psoriasis in adult patients, with studies showing superiority to adalimumab, ustekinumab, and secukinumab.^4^ Here, we present, to the best of our knowledge, the first case of scalp psoriasis resistant to ixekizumab treatment, rapidly and successfully treated with bimekizumab.

## Case report

A 28-year-old female patient was referred to our department in July 2022 because of the presence of erythematous and scaling plaques on the scalp associated with intense itch. On clinical examination, she also showed edema of the second finger of the left hand associated with impaired motility. She had no comorbidities other than obesity (body mass index of 35 kg/m^2^). She has been experiencing plaque-type psoriasis for 10 years and PsA for 5 years. She had been previously treated with cyclosporine and methotrexate for 6 months and 1 year, respectively, without significantimprovement. She therefore started ixekizumab treatment in 2018 reaching complete skin clearance and PsA optimal control. She continued ixekizumab with great results for 4 years until erythematous and scaling plaques on the scalp and edema of the second finger of the left hand appeared, worsening in the following 2 months.

**Fig 1:**
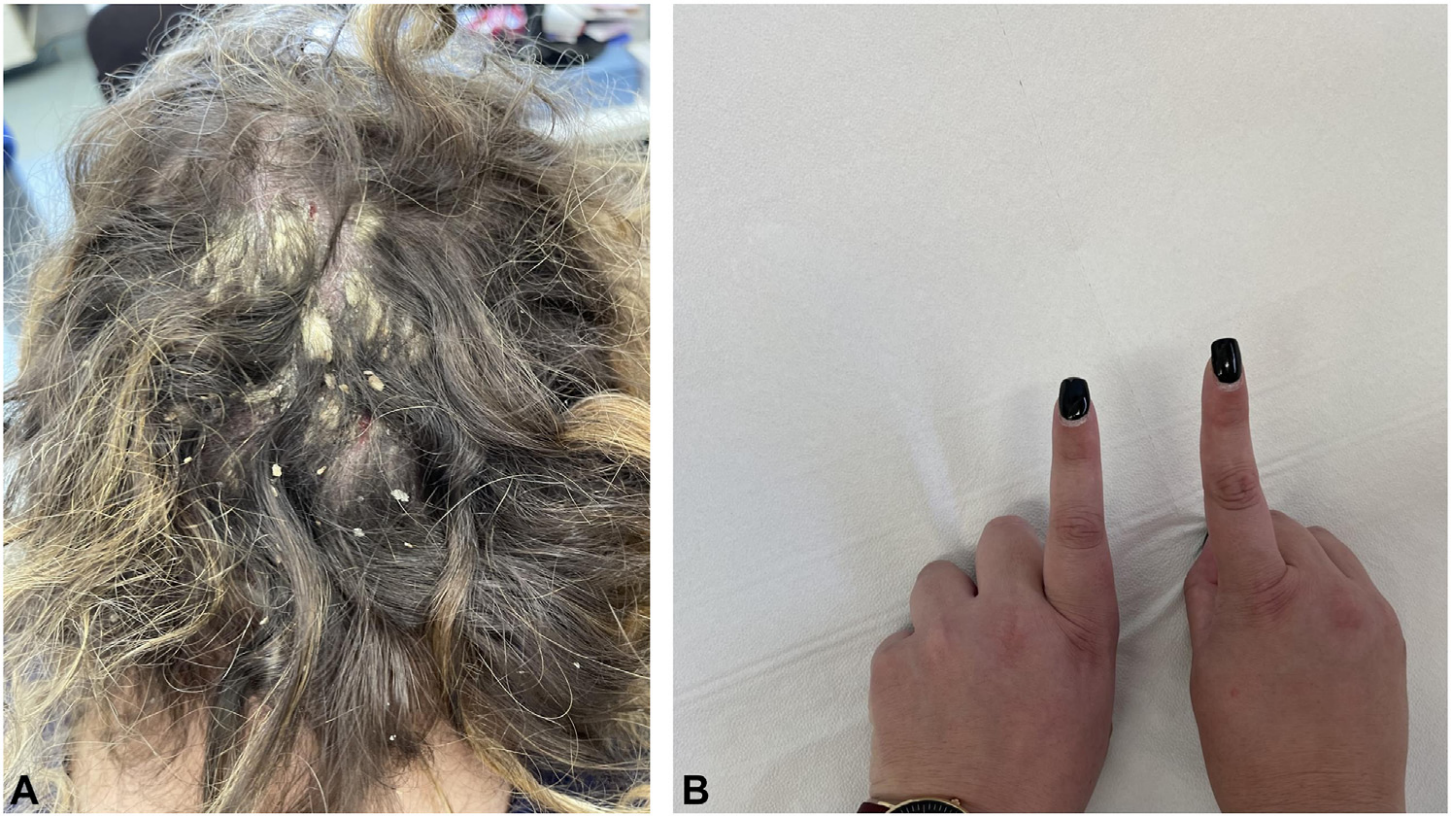
**A,** Erythematous and scaling plaques on the patient’s scalp. **B,** Swelling of the second finger of the left hand between the metacarpophalangeal and interphalangeal joints (sausage digit).

**Fig 2:**
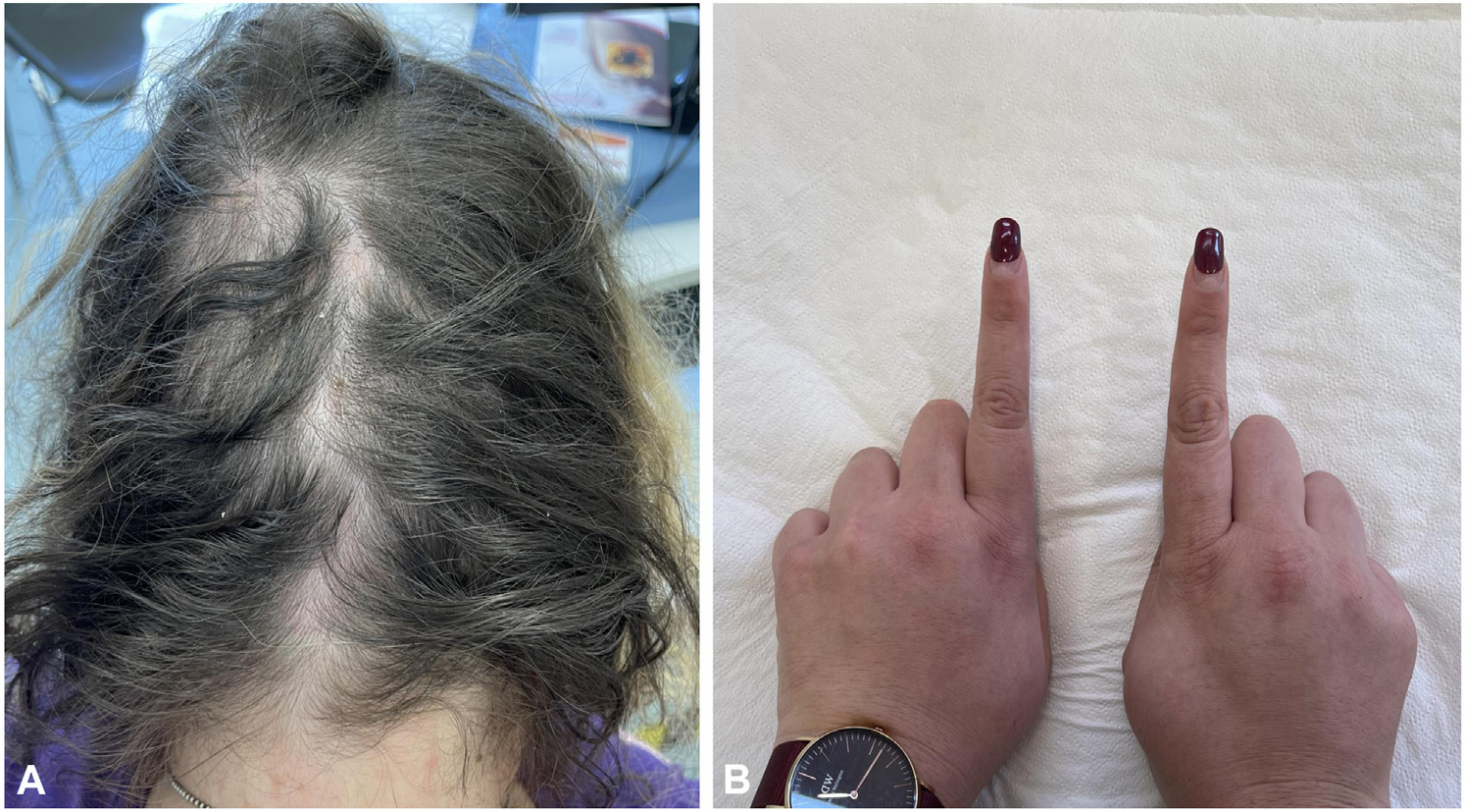
**A,** Complete regression of scalp lesions at week 4. **B,** Reduction in swelling of the second finger of the left hand at week 4.

Dermatologic examination displayed thick hyperkeratotic plaques associated with erythema and desquamation localized on the scalp (Psoriasis Area Severity Index: 10, Psoriasis Scalp Severity Index [PSSI]: 4, and body surface area: 5%; Fig 1, *A*). Blood examination revealed elevated C-reactive protein (5.3 mg/dL vs a normal value of <0.50 mg/dL). The patient also presented with swelling of the second finger of the left hand between the metacarpophalangeal and interphalangeal joints (sausage digit, Fig 1, *B*). Ultrasound examination showed inflammation of the soft tissues supporting the diagnosis of dactylitis. Considering the severe impact on the quality of life as well as the need for a prompt and rapid resolution of both psoriasis lesions and PsA treatment with standard dose bimekizumab was started. Indeed, anti–IL-17 are characterized as the fastest biologic drugs and bimekizumab blocks both IL-17A and IL-17F, unlike ixekizumab which blocks IL-17A only.^4^^,^^5^ Bimekizumab was administered by subcutaneous injections at the standard dose of 320 mg at week 0, 4, 8, 12, and 16 and led to a complete remission (Psoriasis Area Severity Index 100) and PSSI score 0 as early as week 4 (Fig 2, *A*) maintained up to week 12 showing rapidity and effectiveness even on a difficult-to-treat areas, such as the scalp.^2^ Dactylitis also showed improvement on clinical examination and ultrasound with reduction of enthesitis (Fig 2, *B*). The patient reported no adverse event.

## Discussion

Scalp psoriasis is linked to severe discomfort and impairment of quality of life given the associated symptoms, such as scaling and pruritus, the location in a highly visible area and in some cases even the development of psoriatic alopecia.^6^^,^^7^ Therefore, it requires effective and prompt treatment so as to reduce discomfort and improve patients' quality of life. A retrospective, observational, single-center, real-life study on 127 patients affected by scalp psoriasis compared the efficacy of anti–IL-23 drugs (guselkumab, tildrakizumab, and risankizumab) and anti–IL-17 or anti–IL-17RA biologics (secukinumab, ixekizumab, and brodalumab).^8^ Results showed that both anti–IL-17 and anti–IL-23 appeared to be effective on scalp psoriasis.^8^ In particular, patients treated with anti–IL-17 drugs reached a faster significant reduction of the lesions. Bimekizumab has been recently approved for the treatment of moderate-to-severe plaque psoriasis in adults who are candidates for systemic therapy.^3^ It differs from other anti–IL-17 drugs because it selectively inhibits IL-17F in addition to IL-17A.^3^ Dual inhibition of IL-17A and IL-17F is a highly effective therapeutic option for the treatment of psoriasis, both for naïve patients and for those resistant to previous biologic treatments.^3^^,^^4^ Data from 3 phase III, multicenter, randomized, double-blind, placebo-, and comparator-controlled clinical trials (BE READY, BE SURE, BE VIVID, BE RADIANT) have been published to date, showing bimekizumab superior efficacy over adalimumab, ustekinumab, and secukinumab.^9^, ^10^, ^11^, ^12^ There are data regarding the efficacy of bimekizumab for scalp psoriasis also in clinical trials. In particular, BE VIVID, a 52-week, phase 3, randomized, ustekinumab, and placebo-controlled study included, among the efficacy end points, scalp Investigator’s Global Assessment (IGA) 0/1 response at week 16 in patients with scalp psoriasis at baseline (baseline scalp IGA score of ≥2).^13^ At week 16, bimekizumab-treated patients had a higher IGA response versus ustekinumab and placebo (IGA 0/1: 83.1% vs 65.4% and 11.8%, respectively). To date, no real-life experiences or significant case reports regarding bimekizumab for scalp psoriasis are available in literature because of its very recent approval.

## Conclusion

To our knowledge, we present the first case of concomitant scalp psoriasis and PsA resistant to ixekizumab successfully and rapidly treated with bimekizumab. Our case demonstrated the efficacy of bimekizumab in treating both scalp psoriasis and PsA, presenting a very rapid onset of action, rapidly reaching a PSSI value of 0 at week 4 and maintaining the response until week 12 along with control of PsA symptoms.

Undoubtedly, further studies and data are needed to deepen the knowledge about the efficacy and safety of bimekizumab in real life, especially in multifailure patients and, among them, particularly those who have already experienced therapy with other anti–IL-17.

## Conflicts of interest

None.
